# Congenital peritoneopericardial diaphragmatic hernia in a terrier dog

**Published:** 2014

**Authors:** Reza Kheirandish, Mehdi Saberi, Dariush Vosough, Nasrin Askari

**Affiliations:** 1 *Department of Pathobiology, Faculty of Veterinary Medicine, Shahid Bahonar University of Kerman, Kerman, Iran; *; 2 *Department of Clinical Sciences, Faculty of Veterinary Medicine, Shahid Bahonar University of Kerman, Kerman, Iran.*

**Keywords:** Congenital defect, Diaphragmatic hernia, Dog

## Abstract

A one-month-old male terrier dog was referred in shock status with a history of anorexia, tachypnea, abdominal distention and progressive weight loss. Auscultation of right side of the lungs found enhanced respiratory noises. The thorough auscultation of the opposite side of the chest revealed the presence of typical intestinal sounds. Cardiac auscultation revealed muffled heart sounds and a diminished palpable precordial cardiac impulse was evident. The radiograph showed the presence of gas within the bowel in abrupt contrast to the adjacent structures of soft tissue opacity. Conservative treatment was failed and the animal died. At necropsy, cranial displacement of abdominal viscera into the pericardial sac was seen. A definitive diagnosis of peritoneopericardial diaphragmatic hernia was made. Although congenital pericardial diseases are rare in dogs, awareness of the clinical manifestation of these kinds of defects combined with early use of available imaging modalities can yield a preoperative diagnosis.

## Introduction

Congenital pericardial diseases are rare in dogs and cats and most of them have been reported as incidental findings on post-mortem examinations. Peritoneopericardial diaphragmatic hernia (PPDH) is the most common congenital defect involving the pericardium of dogs and cats.^1^ In this anomaly, abdominal contents are herniated into the pericardial sac because of direct communication between the peritoneal and the pericardial cavities. An embryonic development defect of the dorsolateral septum transversum in the so-called sternocostal triangle structure can cause this developmental abnormality.^2^^,^^3^ Other congenital anomalies such as hydrocephalus, umbilical hernias, sternal defects, cranial midline abdominal hernias, abnormal swirling of hair on the ventral abdomen, intra-cardiac defects, and pulmonary vascular can be associated with this anomaly,^4^^-^^6^ whereas, umbilical hernias being the most frequent finding.^3^ Peritoneopericardial diaphragmatic hernia has been reported in dogs, cats, rabbits, a donkey, and a calf.^4^ Generally, Weimaraners are mentioned among the dog breeds that show a predilection for this disorder, as PPDH accounts for 0.5% of their congenital cardiac diseases.^3^ Possible potential causes for PPDH occurrence include malformations and teratogen-induced factors.^7^ Peritoneo-pericardial diaphragmatic hernias are rare but should be included as a differential diagnosis in dogs with an enlarged cardiac silhouette. The main tool for diagnosis of congenital diaphragmatic hernia is radiography, whereas treatment is surgical.^1^^,^^8^^,^^9^ These hernias are also incidental findings at necropsy.^10^^-^^12^ This report describes the post mortem findings, clinical and radiological features of a rare case of peritoneopericardial diaphragmatic hernia in a terrier dog.

## Case Description

**Fig. 1:**
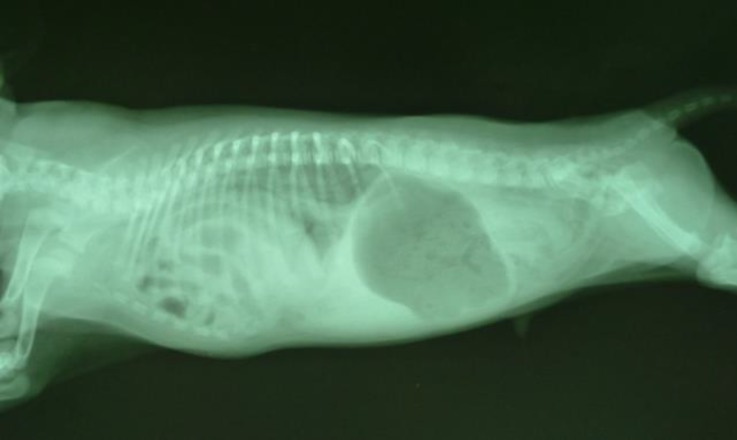
Lateral view. Abdominal organs identified in the pericardial sac: gas and ingesta filled bowel within the thorax

**Fig. 2:**
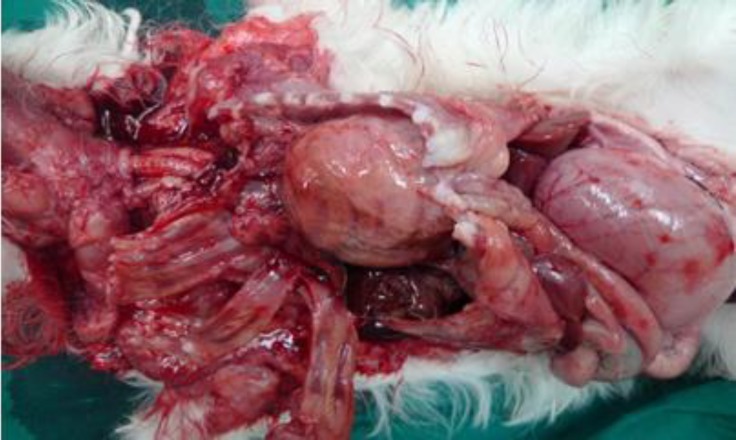
Cranial displacement of abdominal viscera into the pericardial sac was seen, while the pleural space remained intact

**Fig. 3:**
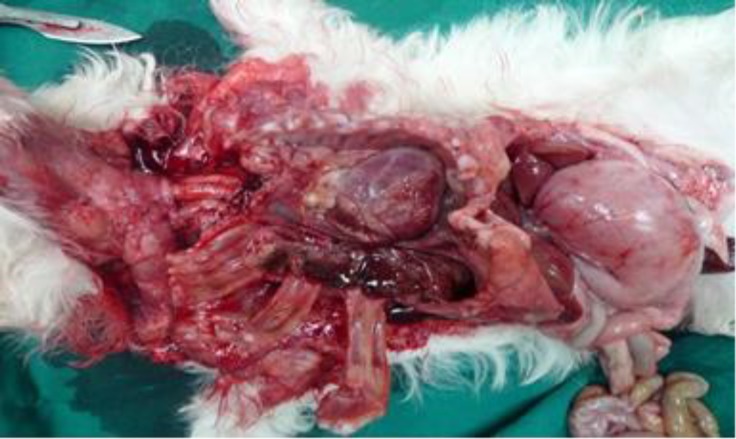
After removal of intestinal loops from hernial sac, there was a markedly enlarge heart

A 1-month-old male terrier dog was referred with a history of anorexia, tachypnea, abdominal distention and lower body-weight gain comparing its littermates. Body temperature, pulse rate, and heart rate were considerably increased and the animal was in shock status. The mucous membranes were slightly cyanotic and auscultation of right side of the lungs found enhanced respiratory noises. The thorough auscultation of the opposite side of the chest revealed the presence of typical intestinal sounds. The evaluation of abdomen was negative for fluid accumulation or palpable mass. Cardiac auscultation revealed muffled heart sounds and a diminished palpable precordial cardiac impulse was evident. Conservative treatment was initiated with intravenous Ringer’s solution and hydrocortisone. Laboratory examination revealed leukocytosis and tendency to left shift to metamyelocytes. Radiological evaluation was performed in lateral projection. The radiograph showed the presence of gas within the bowels in abrupt contrast to the adjacent structures of soft tissue opacity. An indistinguishable outline to the ventral diaphragmatic surface and the caudal silhouette was produced by the communication between two structures (Fig. 1). Despite of conservative treatment, the puppy died due to poor clinical conditions. At necropsy, cranial displacement of abdominal viscera into the pericardial sac was found, while the pleural space was intact .Moderate edema of the intestinal walls was present and a small quantity of serosanguineous fluid was observed. Above all, a markedly enlarged heart into hernial sac was also observed (Figs. 2 and 3). On the basis of this finding, a definitive diagnosis of peritoneopericardial diaphragmatic hernia was made.

## Discussion

Clinical signs of peritoneopericardial diaphragmatic hernia are not specific and depend on the degree and nature of herniation. The most frequent clinical signs involve the digestive tract (vomiting, diarrhea, anorexia, weight loss) and the respiratory system (dyspnea, cough, and wheeze). Abdominal discomfort or swelling, shock and collapse occur less frequently,^13^ whereas, this case was characterized by significant clinical signs. The most part of patients suffering from diaphragmatic peritoneopericardial hernia are identified within the first years of life (48.0%), but a relatively high percentage is identified later, after 8 years of age (6.0%),^3^^,^^13^ and this is partly due to the fact that traumatic events, even late in patient's life, may cause abdominal contents to move into pericardial cavity giving rise to acute clinical signs. However, it should be stressed that this abnormality is never acquired because, unlike in human, the peritoneal and pericardial cavities are not directly connected.^8^ As for the diagnostic imaging techniques employed, the radiological evaluation proved to be decisive for the diagnosis in this case. The hallmark radiographic sign indicating the bowels presence in pericardial sac was the evidence of gas identified in the herniated intestinal loops as visualized on the radiogram. Although this case was too advanced for treatment, surgical repair of the defect is the recommended treatment if other congenital defects do not coexist.^13^^-^^15^ In summary, the low incidence of peritoneopericardial diaphragmatic hernia should not be the cause of omission of the condition in differential diagnosis when consistent clinical signs are present. Thus, reporting similar cases could yield useful information about the diagnosis and treatment of this anomaly.
